# The Role of Novel Imaging and Biofluid Biomarkers in Traumatic Axonal Injury: An Updated Review

**DOI:** 10.3390/biomedicines11082312

**Published:** 2023-08-20

**Authors:** Marios Lampros, Nikolaos Vlachos, Parmenion P. Tsitsopoulos, Anastasia K. Zikou, Maria I. Argyropoulou, Spyridon Voulgaris, George A. Alexiou

**Affiliations:** 1 Department of Neurosurgery, School of Medicine, University of Ioannina, St. Niarhou Avenue, 45500 Ioannina, Greece; marioslampros@gmail.com (M.L.); nickosvlachos826@gmail.com (N.V.); svoulgar@uoi.gr (S.V.); 2 Department of Neurosurgery, Hippokratio General Hospital, Aristotle University of Thessaloniki School of Medicine, 54942 Thessaloniki, Greece; ptsitsopoulos@auth.gr; 3 Department of Radiology, University of Ioannina, 45110 Ioannina, Greece; anzikou@uoi.gr (A.K.Z.); margyrop@uoi.gr (M.I.A.)

**Keywords:** traumatic brain injury, axonal injury, advanced imaging, biomarkers, outcome

## Abstract

Traumatic brain injury (TBI) is a leading cause of disability worldwide. Traumatic axonal injury (TAI) is a subtype of TBI resulting from high-impact forces that cause shearing and/or stretching of the axonal fibers in white matter tracts. It is present in almost half of cases of severe TBI and frequently associated with poor functional outcomes. Axonal injury results from axonotomy due to mechanical forces and the activation of a biochemical cascade that induces the activation of proteases. It occurs at a cellular level; hence, conventional imaging modalities often fail to display TAI lesions. However, the advent of novel imaging modalities, such as functional magnetic resonance imaging and fiber tractography, has significantly improved the detection and characteristics of TAI. Furthermore, the significance of several fluid and structural biomarkers has also been researched, while the contribution of omics in the detection of novel biomarkers is currently under investigation. In the present review, we discuss the role of imaging modalities and potential biomarkers in diagnosing, classifying, and predicting the outcome in patients with TAI.

## 1. Introduction

Traumatic brain injury (TBI) is a leading cause of disability and death, with approximately 70 million people affected each year globally [1]. Traumatic axonal injury (TAI) is a subtype of TBI resulting from high-impact shearing forces, usually rapid acceleration and/or deceleration like those observed in motor vehicle collisions, that cause shearing and/or stretching of axonal fibers in white matter tracts [1,2]. It is present in almost half of the cases of severe TBI, with patients often exhibiting prolonged unconsciousness despite the absence of extensive intracranial traumatic lesions [2]. Axonal injury is also related to long-term neurobehavioral symptoms, mood disorders, and memory impairment, and symptoms of upper motor injury may be present due to damage in the corticospinal tracts [1,2]. 

Even though the previously utilized term, diffuse axonal injury (DAI), suggests widespread axonal damage, this damage is of variable severity. The wide application of novel imaging modalities has shown that milder axonal damage is present in patients with concussion, with the level of axonal damage linked to long-term functional outcomes and neurocognitive impairment [1,2,3]. Additionally, the spatial distribution of axonal lesions is not homogenous, but there is a predilection for specific locations, usually the white matter disjunction, the corpus callosum, and the brain stem [2,3]. Thus, in order to overcome these restrictions, there is a tendency to replace the term DAI with the term TAI [3,4]. 

Although the first reports of widespread white matter injury were described around 50 years ago [5,6,7], the term “diffuse axonal injury” was identified as a distinct clinical and pathological entity in 1981 by Adams and colleagues [8,9,10,11]. At the end of the 1990s, Adams et al. proposed the currently utilized classification based on the observation that the clinical severity of TAI correlates with some clusters of histopathological findings. In particular, they identified three grades of injury: Grade 1, with predominant white matter injury in the white matter of cerebral hemispheres; Grade 2, with a focal injury in the corpus callosum, additionally to the findings of Grade 1; and Grade 3, with a focal lesion in the brainstem, additionally to findings of Grade 2 [10]. 

The advent of novel histopathological and imaging diagnostic tools has increased our understanding of the molecular and biochemical mechanisms of TAI. Following TBI, it is clear that axonotomy does not result from primary mechanical shearing of axons during trauma but is a consequence of a complex secondary biochemical cascade that has yet to be fully elucidated [12]. Moreover, the introduction of functional MRI techniques, such as diffusion tensor imaging, has led to drastic improvements in the detection of axonal trauma in the living brain and its clinical correlations. 

In the present narrative review, we summarize the current literature on the role of imaging modalities as well as fluid and structural biomarkers in the diagnosis, management, and outcome of patients with TAI. 

## 2. Search Protocol

MEDLINE/PubMed, Scopus, and Google Scholar were the main databases utilized for the research. Moreover, the relevant articles from the reference lists of selected publications were included if relevant. The search algorithm in PubMed/MEDLINE included the following: (Traumatic axonal injury or diffuse axonal injury) and (biomarkers or imaging). A similar modified algorithm was utilized in the SCOPUS and Google Scholar databases. The last research was performed on 15 May 2023.

## 3. Imaging 

Currently, there are no established clinical and imaging criteria for diagnosing TAI. In clinical practice, the diagnosis is established by means of co-evaluation of clinical and imaging features. Loss of consciousness or a low Glasgow Coma Scale (GCS) score (under or equal to eight) for more than six hours in a patient with head trauma, along with the presence of multiple small white matter lesions in the grey–white matter disjunction are characteristics that have been considered highly indicative of TAI [4,13]. However, this definition has several limitations. First, the time limit of 6 h in differentiating concussion from TAI originated from animal experiments around 30 years ago [4]. Moreover, CT scan is the primary imaging modality performed in patients with TBI, which has a very low sensitivity in detecting small white matter lesions [4,13,14]. Additionally, CT is not sensitive to the detection of non-hemorrhagic (petechiae) TAI lesions, which account for approximately 80–90% of all TAI. Furthermore, not all patients with axonal lesions, especially those with focal rather than diffuse, present with low GCS score. Lastly, axonal lesions can be observed in patients with mild or moderate TBI, such as in athletes in contact sports (e.g., boxers) [4,14,15]. 

**Figure 1:**
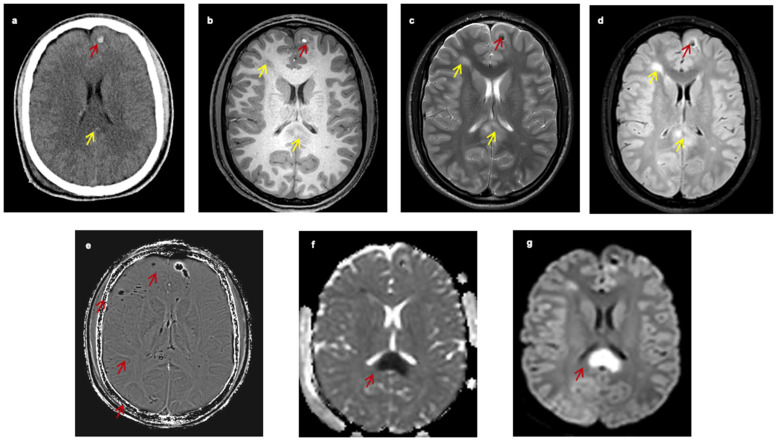
17-Year-old male with traumatic axonal injury (unpublished data) (**a**) CT scan on admission shows hyperdense foci (microhaemorrhage) in the gray–white matter junction of the left frontal lobe (red arrow) and hypodensity of the splenium of the corpus callosum (yellow arrow). (**b**) MRI, three days later, axial T1WI shows a hyperintense lesion at the gray–white matter junction of the left frontal lobe compatible with subacute hemorrhage (red arrow) and hypointense lesions in the deep white matter of the right frontal lobe and the slpenium (yellow arrow). (**c**) MRI axial T2WI, and (**d**) FLAIR images show the left frontal lobe lesion with low signal (red arrow) and the lesions of the deep white matter of the right frontal lobe and the splenium with high signal intensity (yellow arrow). (**e**) MRI axial SWI sequence shows multiple low signal intensity lesions compatible with haemorrhage mainly located in the gray–white matter junction of fronto-occipital lobes and the right side of the splenium of corpus callosum (red arrow). (**f**) DWI image (**g**) and ADC parametric map reveal restricted diffusion within the splenium of the corpus callosum (red arrows), compatible with cytotoxic oedema. Key: CT, computed tomography; MRI, magnetic resonance imaging; WI, weighted imaging; SWI, susceptibility-weighted imaging; DWI, diffusion-weighted imaging.

MRI is a far more sensitive imaging modality than CT scan for detecting small white matter lesions, but it is infrequently available in the acute phase [3,16,17,18]. Despite the progress that has been achieved in detecting TAI lesions, the only definitive way to confirm the diagnosis is via brain biopsy or post-mortem histopathological evaluation [4]. Figure 1 presents the appearance of TAI lesions in different imaging modalities of one patient with TAI (Figure 1).

### 3.1. Current Modalities

#### 3.1.1. CT Scan

An unenhanced CT scan is the imaging modality of choice for the initial evaluation of patients with TBI [4]. In the acute phase, the primary aim of the CT scan is to detect the presence of hemorrhagic lesions or contusions and evaluate the risk of brain herniation. CT scan findings and clinical presentation will determine the need for acute neurosurgical intervention [4,14,15,19,20,21,22]. Because patients with TAI usually present with LOC and low GCS on admission, they almost always undergo an unenhanced CT scan [4]. Due to local capillary damage, axonal lesions are usually not detectable on CT scans if they are not accompanied by significant hemorrhagic elements, usually seen as hyperdense small petechiae. Hence, the hallmark of TAI on CT scans is the presence of diffuse bihemispheric petechiae lesions in the gray–white matter interface (Figure 1a). The CT will detect approximately 20% of these lesions as small hypointense (under 1–2 cm) areas. Since TAI lesions are usually underseen on CT, it has been suggested that their presence is related to extensive brain damage and poor outcome, but this has been recently questioned [14,19]. Despite the disadvantage of CT for being insensitive in detecting TAI lesions, these findings can be indirectly used to diagnose TAI. In a recent systematic review of the literature, Figueira et al. found that TAI lesions are usually accompanied by intraventricular or subarachnoid hemorrhage. Specifically, the presence and the extent of intraventricular or subarachnoid hemorrhage positively correlate with the existence and severity of TAI. At the same time, the absence of these hemorrhagic lesions rules out significant axonal injury [19]. 

#### 3.1.2. MRI Magnetic Resonance Imaging (MRI)

Conventional MRI is approximately two to three times more sensitive than CT in detecting TAI lesions [13]. However, the sensitivity is lower for the detection of non-hemorrhagic lesions. The signal is usually hyperintense in T2 and FLAIR sequences, while the signal varies in T1 sequences; it is low when the lesion is non-hemorrhagic or high when the lesion has hemorrhagic components (Figure 1b–d) [13,23]. The histopathological grading proposed by Adams et al. [23] has also been utilized in the imaging classification of these lesions. In 2017, Hamdeh et al. [13] proposed an extended classification of the typical three-grade system based on the observation that TAI lesions in the substantia nigra and the tegmentum were independent risk factors for poor outcome and hence represented a distinct fourth grade. Except for the conventional MRI sequences, other MRI sequences such as susceptibility-weighted imaging (SWI), diffusion-weighted imaging (DWI), and diffusion tensor imaging (DTI) have substantially improved the diagnostic sensitivity of MRI to detect TAI lesions [24]. 

### 3.2. Novel Techniques

#### 3.2.1. Gradient Recalled Echo-T2* and Susceptibility Weighted Imaging Sequences (SWI)

**Figure 2:**
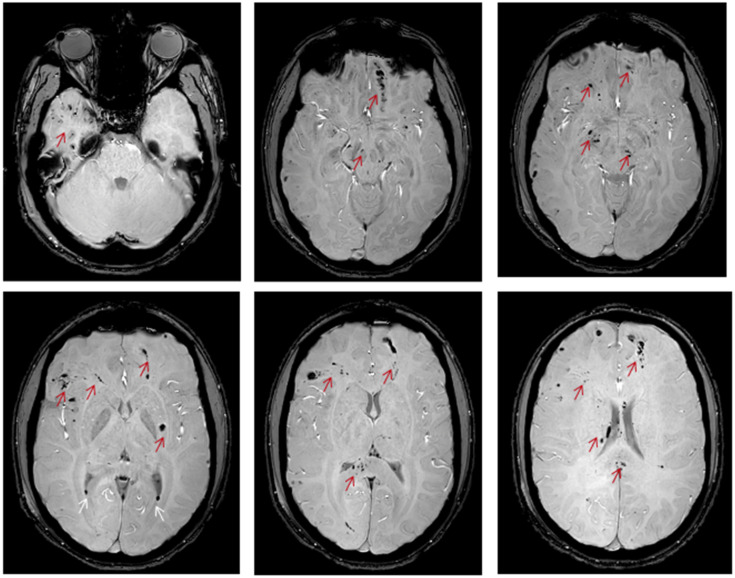
MRI of a 17-Year-old male with traumatic axonal injury, three days post traumatic brain injury (unpublished data), axial plane, SWI sequence shows multiple “dark spots’’ suggestive of haemorrhagic lesions mainly located in the gray–white matter junction of the right temporal, the fronto-occipital lobes and the right side of the splenium of corpus callosum, the midbrain and the left basal ganglia (red arrow). Key: MRI, magnetic resonance imaging; SWI, susceptibility weighted images.

When TAI lesions have hemorrhagic components, techniques that detect blood breakdown products, such as the conventional gradient recalled echo (GRE)-T2* and the advanced SWI (Figure 1e and Figure 2), are preferred. The SWI enhances the contrast of GRE-T2* sequences by combining T2*-magnitude and phase images [25]. Hence, the SWI can detect smaller lesions. In a study of seven young patients with TAI, Tong et al. found that SWI could detect ten times more hemorrhagic lesions than GRE-T2* (1038 vs. 162) [25]. In a larger study including 40 young patients, it was found that the number and volume of hemorrhagic lesions depicted in SWI significantly correlated with patients’ functional outcomes [26]. However, these techniques can only distinguish TAI lesions in the sub-acute or chronic phase [25] because they detect blood breakdown products and hence can mainly support the diagnosis of TAI in cases of diagnostic doubt.

#### 3.2.2. Diffusion-Weighted Imaging 

In highly organized structures that contain macromolecules such as proteins or membranes, free diffusion of the water molecules is restricted [18]. Diffusion MRI creates contrast MRI images by detecting the degree of random motion of water molecules [18,27,28]. It is a very sensitive technique in detecting brain lesions within the first 3 h after the incident, contrary to the conventional CT and MRI techniques which require at least 8–12 h to visualize the infarct [29]. Ischemia generates cellular cytotoxic edema, contrasting with the surrounding non-ischemic tissues. Similarly, after TAI, the shearing forces cause local edema and disruption of the axonal architecture, thus resulting in alterations in diffusion images (Figure 1f,g). 

In a study of 19 patients with TAI, Huisman et al. found that DWI was more sensitive in detecting TAI lesions than FLAIR and T2*. The majority of lesions had restricted diffusion. Interestingly, the diagnostic ability of DWI was not limited to non-hemorrhagic lesions, which reflect most TAI lesions [18]. The degree of water molecule diffusion is quantified by using the apparent diffusion coefficient (ADC), and parametric maps assigning each voxel to an ADC value can be created. Hergan et al. classified the TAI lesions based on their DWI and ADC parametric map patterns [27]. They observed three types of TAI lesions: type 1 lesions (DWI and ADC hyperintense) that reflect vasogenic edema, type 2 (DWI hyperintense and ADC hypointense) that reflect cytotoxic edema, and type 3 hemorrhagic lesions with variable signal intensity depending on the stage of hemorrhage. However, their distinct prognostic role remains uncertain.

DWI is a promising method for diagnosing TAI and may allow fast detection of axonal lesions in the acute phase of TBI. Moreover, DWI can be performed within a few minutes and will not delay further management of trauma patients. Indeed, it is the only MRI technique that can be used during the first hours following TAI [18]. Contrary to diffusion tensor imaging (DTI), DWI has a lower duration and may be used in hemodynamically stable TBI patients in cases where axonal lesions are suspected [18,27]. 

#### 3.2.3. Diffusion Tensor Imaging (DTI) and Fiber Tractography

In white matter tracts, water diffusion is more restricted perpendicularly (radial diffusion) than parallel (axial diffusion) to the long axis of the tract. This motion preference of water molecules in a particular axis is called anisotropy and depends on several parameters, such as cell size, composition, and axonal alignment. Hence, the degree of anisotropy measured by DTI can offer important information regarding the microscopic composition of the axons. 

Arfanakis et al. [30] performed DTI in five patients with mild TBI. They detected a focal reduction in functional anisotropy (FA) in white matter tracts compared to the contralateral hemisphere, which was not observed in controls. Similarly, Inglese et al. found that FA was reduced in patients with mild TBI. This reduction was more significant in brain areas vulnerable to TAI, such as the corpus callosum and the internal capsule [17]. These results indicate that multifocal axonal injuries can be observed in the wide spectrum of TBI and not only in severe TBI cases. DTI data can be used to create a three-dimensional colored reconstruction of the fibers. The color of the fibers represents the water molecules’ preferred motion direction, while the intensity of the color represents the degree of anisotropy. Several studies support that DTI and fiber tractography can detect axonal injuries that are otherwise not detectable in conventional MRI [31,32,33,34]. Even in the subacute stage, approximately within two weeks from trauma, the amount of damage in the fiber tracts can correlate with the patient’s long-term functional outcome [34]. Due to the unique ability to visualize axonal anatomy, these techniques are very promising in detecting axonal injuries while allowing for a more precise correlation between the site of the lesions and the neurocognitive outcome [30,33].

Myelin water imaging is another novel technique that can detect quantificational changes in the myelin’s water after TBI. The latter is performed by visualizing the short transverse relaxation time component (ViSTa) technique [35,36]. This complex technique refers to a multi-component T2 relaxation sequence in which the short T2 sequence represents the myelin water. In preliminary reports, post-mortem and animal studies have shown that a significant degree of traumatic demyelination can be observed after TBI [35,36]. At the same time, the inadequate remyelination of injured axons is linked with less favorable functional outcomes. Preliminary data support that reducing myelin’s water fraction is a potential imaging biomarker of injury severity and long-term functional outcome [35,36,37].

These techniques are usually performed within a few days following TBI since the patients should be hemodynamically stable and the procedure lasts longer (30–60 min) [30,31,32,33]. The ViSTa technique is currently experimental and its efficacy in the diagnosis of TAI lesions will need to be confirmed by future studies.

#### 3.2.4. Magnetic Resonance Spectroscopy (MRS)

MRS is another advanced MRI technique that can detect neurochemical alterations in the composition of axons. In particular, it can detect the levels of a brain-specific metabolite and marker of preserved axonal integrity, N-acetyl aspartate (NAA). After TBI, NAA levels are decreased, and subsequently, the ratio of NAA/creatine and NAA/choline is decreased, while the ratio of choline/creatinine is increased [38,39]. In a study of 40 children with TBI, Holshouser et al. observed these ratio alterations in children with normal MRI and poor functional outcomes, detecting axonal injuries that were non-detectable in normal MRI [38]. Hence, the MRS could be used as another imaging biomarker for the detection of axonal injuries in children with suspicion of TAI with negative MRI, while recovery of these ratios in the follow-up MRI may be associated with a more favorable outcome [3]. Similarly to DTI and fiber tractography, these techniques are performed when the patient has been stabilized, usually in the days or weeks following trauma [38,39].

#### 3.2.5. Positron Emission Tomography

Most patients with TAI will develop some form of cognitive dysfunction. Scheid et al. suggested that all patients with TAI will have an impairment of at least one cognitive subfunction, with memory and executive dysfunction most frequently affected [40]. Several functional imaging studies have noted that this neurocognitive decline is associated with focal neuronal damage in specific brain locations. Nakashima et al. utilized 18-fluorodeoxyglucose–positron-emission tomography (FDG-PET) to study brain metabolism in 12 patients with long-term neurocognitive impairment following TAI compared to healthy controls. A significant reduction was noted in the cingulate gyrus, but the specific location of hypometabolism was different in each patient [41]. This regional reduction in glucose metabolism compared to controls was also observed in another study with FDG-PET, where the reduction was again prominent in the cingulate gyrus [42].

Flumazenil is a well-known antidote for benzodiazepine poisoning that acts as an antagonist of GABA receptors. Kawai et al. utilized the radiotracer flumazenil (11C-flumazenil) and studied its presence in neurons with PET scans in patients with diffuse TBI-associated neurocognitive deficits vs. normal controls. They observed a reduced uptake in the cingulate gyrus, medial frontal cortex, and thalamus, indicating that focal axonal injury in these locations could be the reason behind neurocognitive impairment [43]. However, in another FDG-PET study in rat models with diffuse axonal study, Li et al. observed that hypometabolism in the rat hippocampus in the acute phase of trauma was linked with long-term learning dysfunction [44]. Finally, in a PET imaging study in patients with repeated sport-related contusions, which are vulnerable to axonal injuries, Maklund et al. noted an increased aggregation of tau protein via the [18F] THK5317 tracer in the corpus callosum compared to normal controls [45]. 

In summary, the above studies have shown that neurocognitive impairment in patients with TAI is linked with lesions in parts of the limbic system [41,42,43,44,45]. The limbic system is a group of brain structures, including the cingulate gyrus, amygdala, thalamus, and frontal cortex, that are highly implicated in neurocognition as well as behavioral and emotional responses [41,42,43]. Hence, since lesions in these anatomic structures are found in patients with TAI or other types of TBI, they could possibly explain the neurocognitive impairment observed in patients with DAI. PET is a promising technique that may allow an in-depth study of the long-term neurocognitive consequences of TBI and correlate them with specific anatomic brain regions that have been injured [42,43,44,45]. However, it is not currently used in the acute and sub-acute phases of TBI, and is mainly an experimental procedure.

### 3.3. Summary of Novel Imaging Techniques

Even though MRI remains the cornerstone of TAI imaging diagnosis, the introduction of more axonal-specific imaging techniques is expected to add useful diagnostic information in cases where the clinical diagnosis and imaging of TAI is challenging [4,13,15]. Each technique has its advantages for different trauma phases and types of TAI lesions (e.g., hemorrhagic or non-hemorrhagic) [25]. DWI is the only technique that is useful and available to be performed within the first few hours following trauma [18]. DTI, fiber tractography, and MRS are typically performed within the first few days following TBI, typically in addition to the conventional MRI, while (GRE)-T2* and SWI are useful in the first few weeks following TAI in cases of hemorrhagic lesions to detect the blood breakdown products in areas of grey–white matter disjunction [3,4,18]. Most of the abovementioned MRI sequences and techniques are already widely used in other fields of neuroimaging, i.e., fiber tractography and MRS in neuro-oncology, and, hence, they can be easily utilized to explore their full potential in neurotrauma diagnosis. 

## 4. Biofluid Biomarkers of TAI

### 4.1. Introduction 

In the past two decades, there has been increasing interest in developing the role of potential biomarkers in predicting the presence of specific brain lesions and the short- and long-term outcomes of patients with TBI. A wide range of brain biomarkers has been reported in the literature, including neuronal or axonal (e.g., neuron-specific enolase, UCH-L1, Tau, Neurofilament), astroglial (S100β, GFAP), and other less brain-specific biomarkers such as D-dimers, creatine phosphokinase, and neutrophil to lymphocyte ratios [46,47,48]. Regarding their use in TAI, only a limited number of studies are available, and even fewer in pediatric TAI. A summary of the human studies focusing on the role of TBI serum biomarkers in patients with TAI is presented in Table 1 [49,50]. 

Following TAI, axonal injury can be caused primarily via direct (mechanical) axonotomy due to the impact of shearing forces, but the damage that occurs is mainly secondary due to the activation of intracellular proteases that cause degradation of the axonal micromolecules. Specifically, oxidative stress increases the influx of Ca^+2^ and induces a protease (calpain)-mediated cleavage of axonal molecules such as neurofilaments, spectrin, Tau, beta-APP, and myelin-based proteins (MBP) [12,49,50,51,52]. 

### 4.2. Fluid and Structural Biomarkers for TAI

#### NFL (Axonal Biomarker)

Neurofilaments are heteropolymers that contain three different chains: a heavy chain (NfH), a medium chain (NfM), and a light chain (NFL). The neurofilament light chain (NFL) is an intermediate cellular filament, part of the axonal cytoskeleton involved in axonal stability, axonal transport, and determination of axon diameter. In patients with axonal injury, the NFL can be directly (mechanically) or secondarily damaged via TBI-induced phosphorylation that alters axonal stability and is released into the surrounding tissues, CSF, and blood [51,53,54]. It has been found that following TBI, the NFL is increased up to 500-fold in CSF at 1 h post injury and up to 40.000 at 6 h post injury. Hence, the CSF concentrations of NFL have been proposed as a potential biomarker to assess the severity of TAI. However, the measurement of NFL concentrations in CSF is an invasive procedure that can potentially risk brain herniation in patients with TBI [54]. In the blood, this increase is less significant, partially due to the blood–brain barrier (BBB) that impairs the transfer of macromolecules [54]. 

Ljungqvist et al. studied nine adult patients with TAI and found that their serum NFL concentration was raised 40-fold compared to controls. Except for the NFL, the neurofilament heavy chain (NFH), especially the calpain-resistant, phosphorylated NFH, has also been found to be significantly correlated with patient outcome and the presence of imaging findings [53]. In particular, phosphorylation of NFH in the lysine–serine–proline repeats (KSP), a repeated sequence of about seven amino acids in the sequence lysine–serine–proline that are highly specific for axonal detection because they are not detected in dendritic and pericaryal NFH. Hence, its concentration is expected to increase in cases of diffuse axonal injury [51]. The levels of pNFH were found to be positively correlated (*p* = 0.004) with the presence of TAI findings in the initial CT of children with TBI [53]. 

In the coming years, neurofilaments, due to their physiological role as part of the axonal skeleton, are expected to be one of the most studied biomarkers for TAI.

### 4.3. Tau (Axonal Biomarker)

Tau is a microtubule-associated protein involved in the stability of axonal microtubules. Following axonal injury, the Tau protein is cleaved by calpain proteases, resulting in the cleaved microtubule-associated (c-Tau) form that can be measured in the serum. At the same time, the TBI induces hyperphosphorylation of Tau (p-Tau), resulting in the aggregation of microtubules that form insoluble tangles [55,56,57]. Tomita et al. found that the blood concentration of total Tau was higher in TBI patients with TAI vs. non-TAI, with 74.1% and 69.2% sensitivity and specificity, respectively. However, Tau levels did not correlate with the patient’s outcome in a significant manner (*p* = 0.19) [58]. Except for TAI, total Tau levels rapidly increase in the blood and CSF of athletes performing contact sports (e.g., boxers) that have experienced some degree of axonal injury. Hence, the increased Tau levels in these patients could be partially attributed to axonal injury [3,59,60]. In an experimental study with rats that sustained high-impact spinal cord compression or complete transaction of white matter tracts of the spinal cord, the total Tau levels significantly increased 550-fold in the CSF at the first post-injury day compared to naive rats, while the levels of p-tau were elevated 7-fold. Finally, a significant increase in the serum levels of total tau (1.2-fold) and p-tau (7-fold) compared to naive rats was noted [61]. Total Tau and pTau levels have also been suggested as a potential biomarker for the diagnosis and progress of several neurodegenerative diseases, including Alzheimer’s disease. However, the measurement of Tau/p-Tau levels in biological samples is a challenging procedure, mainly because Tau/p-Tau concentrations are very low, and their detection along with the study of their kinetics requires novel methods such as with the use of digital ELISA [61]. 

Tau is a protein that plays a significant role in axonal stability; hence, it is a promising biomarker for detecting axonal injuries. Despite the moderate sensitivity and specificity observed in some studies, the development of ultrasensitive detectors that allow its measurement and kinetics in very low concentrations is a favorable factor for the conduction of future studies regarding its role as a potential biomarker for axonal injury.

### 4.4. S100β (Glial Biomarker) and NSE (Neuronal Biomarker)

S100β is an astroglial molecule with currently unclear physiological significance that is significantly elevated following TBI. S100β is a sensitive molecule that is used to rule out significant brain injury, and it has been included in the Scandinavian guidelines for determining the need to undergo a CT scan in patients with mild TBI [62,63]. NSE is a glycolytic enzyme found in increased concentrations in neurons and has been widely studied as a potential biomarker of TBI. 

In a study of 28 patients with TAI, both S100β and NSE significantly correlated with long-term outcome, measured on the Glasgow Outcome scale. The levels of S100β reached a maximum level after 6 h, while the levels of NSE remained stable within the first three days [64]. In another study, Abbasi et al. found that in patients with suspected TAI in a CT scan, the mean levels of S100β were significantly higher (632.18 ± 516.1 ng/dL) compared to those with normal CT scan or other intracranial traumatic findings, namely bone fractures and intracranial hemorrhage [65]. 

However, these biomarkers are not axonal-specific and are expressed from cells outside the CNS [3]. Hence, only limited studies exist regarding their use in TAI, while their increase could correlate with other extra-cranial or brain injuries.

**Table 1:** Summary of human studies focusing on the role of TBI serum biomarkers in patients with traumatic axonal injury. n/s, not specified. ELISA, enzyme-linked immunosorbent assay. SIMOA, single molecular assay, TAI, traumatic axonal injury.

Author	Year	Biomarker	Number of Patients	Increase in TAI Patients	Control Group	Method Utilized	Sensitivity	Specificity	Cut Off
Ljungqvist et al. [54]	2017	Neurofilament light chain	9	40-fold	Healthy adults	SIMOA	n/s	n/s	n/s
Tomita et al. [58]	2019	Total Tau	40	20-fold	TBI patients with non-TAI lesions	ELISA	74.1%	69.2%	1.5 pg/mL
Abbasi et al. [65]	2014	S100 beta	98	15-fold	TBI patients with non-TAI lesions	ELISA	n/s	n/s	n/s
Chabok et al. [64]	2012	S100 beta	28	Increased (n/s)	TAI patients with good outcome	ELISA	88%	100%	49.3 ng/L
Chabok et al. [64]	2012	Neuron specific enolase	28	Increased (n/s)	TAI patients with good outcome	ELISA	n/s	n/s	n/s

### 4.5. Spectrin (Axonal Biomarker)

Spectrin is a cytoskeletal protein that is part of the plasmatic membrane critical for maintaining membrane stability and cell shape. Although the role of spectrin in the plasmatic membrane of red blood cells is well described, its exact contribution to the axonal skeleton’s stability and organization is unclear [66,67,68]. Following TAI, the calpain and caspase three activation results in the proteolytic cleavage of spectrin into spectrin breakdown products (SBDP). The role of SBDP as a potential biomarker in mild TBI in humans has been previously investigated [69]. Regarding its prognostic role in TAI, Siman et al. [70] found that the serum levels of SNTF, a cleavage of αII-spectrin1-1176, could accurately predict long-term brain dysfunction in patients with long post-concussion syndrome, an entity strongly related to axonal injury. Indeed, it has been found that post-concussion syndrome is an entity with clinical, imaging, and histopathological correlation with axonal injury, with many authors reporting that concussion syndrome is in the milder spectrum of TAI [71]. In a recent post-mortem histopathological study, the immunochemistry pattern of spectrin with beta-amyloid precursor protein (β-APP) was found to be a surrogate marker of axonal injury [72]. Specifically, spectrin and β-APP are both stained in patients with TAI, while in ischemic brain axonal injury, only β-APP is stained. 

Future clinical studies should be performed that include larger patient numbers to explore the role of spectrin as a biomarker in the acute and the sub-acute phase of axonal injuries.

### 4.6. Amyloid Precursor Protein

Amyloid precursor protein (APP) is another membrane protein that is expressed in the cell surface of neurons. Regarding its physiological role, APP is involved in forming synapses and neuronal transport. Following axonal injury, the accumulation of APP in the post-injury-formed axonal bulbs is considered an immunochemistry hallmark of TAI [72,73]. In the axonal bulbs, the APP co-accumulates with enzymes involved in its enzymatic cleavage [74]. The amyloid beta-peptide (Aβ) is derived from the proteolytic cleavage of APP via presenilin. The accumulation of Aβ plaques is a well-known surrogate marker of Alzheimer’s disease, but its role in the pathogenesis of TAI is less well-defined. Interestingly, it has been suggested that patients with repetitive brain injuries are at high risk for the development of Alzheimer’s dementia [75]. 

Currently, studies focusing solely on Aβ following TAI are lacking. In more general reports on Aβ levels after TBI, the levels of Aβ rapidly increased in ventricular CSF in patients with TBI, up to 1173% of Aβ (1-42) in the first week, while the plasma levels remained unchanged [76,77]. 

An ultrasensitive digital ELISA technique, the so-called Single Molecule Arrays (SIMOA), has been utilized to measure plasma and CSF concentrations of Aβ42 and determine their prognostic significance in patients with severe TBI [78]. It was found that CSF levels of Aβ42 were decreased compared to controls, probably indicating Aβ42 amyloid deposition, while plasma levels were elavated shortly after the injury. By contrast, Hossain et al. found that plasma Aβ 40/42 was not significantly different between patients with findings on the CT vs. no findings, as well in patients with different outcomes [79]. Hence, there is no consensus on the kinetics of Aβ42, and more studies should be performed that will consider the potential role of BBB in the permeability of Aβ. 

Similar to Tau/p-Tau, difficulties in measuring and detecting the kinetics of amyloid products are expected to be overcome with the development of more sensitive methods that will allow in-depth analysis and determination of whether or not these biomarkers will be of value in axonal injuries and TBI. Ultrasensitive digital ELISA techniques are an optimal tool for the measurement and the study of kinetics of amyloid peptides. Hence, it is expected that future studies on advanced ELISA or other novel techniques with more patients included will help to clarify the prognostic role of amyloid peptides.

### 4.7. micro RNA (miRNA)

miRNAs are non-coded, small-sized (approximately 20 nucleotides), hairpin-like structures involved in the silencing of messenger RNA (mRNA) at a post-transcriptional level, thus downregulating protein synthesis. miRNAs are coded in the nucleus, and when exported in the cytoplasm, they bind micro RNA-induced silencing complex (miRISC). Next, the miRISC complex is bound on the 3′ end of the targeted mRNA and induces its degradation [80,81,82]. 

Around 70% of miRNAs in the human body are expressed in the nervous system. miRNAs are involved in multiple CNS functions, including the formation of brain synapses, regeneration, and apoptosis [82]. Following TBI, a significant alteration in the expression levels of different miRNAs has been observed, probably reflecting an attempt by the CNS to restore homeostasis. A few miRNAs pass the blood–brain barrier (BBB) and enter the blood circulation as circulatory miRNAs. Thus, several authors suggest that circulatory miRNA can potentially be a diagnostic or prognostic biomarker in different diseases, including cancer, degenerative diseases, and trauma [72,82,83,84]. Regarding their role in TAI, Pinchi et al. found that the expression of three miRNAs (miR21, miR16, miR92) was significantly altered, approximately three times higher, in the brain of patients that died from axonal injury compared to controls, indicating that miRNAs could serve as potential biomarkers of post-mortem diagnosis of brain injury. However, in their study, the role of circulatory miRNA in the grading of living individuals with traumatic axonal injury was not studied. 

Thus, more research should be performed to clarify the exact role of miRNA in the diagnosis and grading of patients with TAI [72]. 

### 4.8. Proteomics 

An alternative “proteomics” approach to identify novel TBI biomarkers has been attempted in recent years. Despite the complexity of proteomic techniques, it is probably an ideal approach to identify biomarkers with strong prognostic significance compared to the “blind” investigation of single molecules as potential biomarkers [85,85,86,87,88]. 

Isobaric tags for relative and absolute quantitation (iTRAQ) is an isobaric labeling method coupled with liquid chromatography and tandem mass spectroscopy (iTRAQ coupled LC-MS/MS) utilized in quantitative proteomics that allows simultaneous comparison of protein profiles from different biological sources [89]. The advantage of this method is that it can detect alterations in the number of specific molecules between different samples, such as among patients with TBI and controls. At the same time, this detection can be enhanced by bioinformatic tools. Hence, it is a potent proteomic technique for identifying potential biomarkers [89,90]. 

Liang et al. used this technique to identify alterations in the expression of molecules in the corpus callosum of rats with TAI. They utilized tissue samples from rat corpus collosum at seven different time points following injury (1, 3, 6,12, 24, 48, and 72 h). Their analysis revealed that among 514 molecules with a significant expression difference, only 14 were present 72 h post injury. Next, they performed bioinformatic analysis to elucidate which of these molecules interfere in the pathogenesis of TAI. They found that peripherin, a cytoskeleton structure, and calsenilin, a calcium ion regulatory protein, may be involved in the pathogenesis or pathophysiology of TAI and can serve as potential biomarkers [91]. According to their bioinformatic analysis, these molecules could be involved in secondary axotomy: calsenilin with calcium-mediated caspase activation and peripherin with the consequent axonal instability. In a similar study in rats with TAI, using brain tissue samples from rats at different time points post injury in the first 7 days, Zhang et al. identified ten differentially expressed proteins, of which four (citrate synthase, synaptosomal-associated protein 25 (Snap25), microtubule-associated protein 1B (MAP1B), and Rho-associated protein kinase 2 (Rock2)) were further validated and expressed by Western Blot and immunochemistry [92]. Finally, in another iTRAQ study in rats, the same authors analyzed plasma biomarkers with TAI at the same time points post injury, and they identified 58 differentially expressed proteins, of which two, glyceraldehyde-3-phosphate dehydrogenase (GAPDH) and hemopexin (Hpx), were found to be involved in the pathogenesis of TAI [93]. 

Despite the lack of clinical studies in humans with TAI, the increased availability of omics will probably allow the conduction of studies in humans with axonal injuries in the near future and provide interesting results. 

## 5. Conclusions

The introduction of advanced MRI sequences, such as DTI and the ViSTa technique, are expected to further improve the diagnosis, accurate grading, and outcome prediction in patients with TAI. These techniques allow a more detailed description of the distribution and extent of axonal pathology. This will probably permit a more extended grading of TAI lesions and a more precise correlation with acute and chronic neurocognitive consequences that are frequently evidenced in these patients. However, most studies have included a small number of patients with TAI, and hence the results from novel imaging techniques should be further validated. At the same time, currently, there are no large series to explore the role of well-known TBI biomarkers that are not specific for axonal damage, such as GFAP and UCH-L1. However, the advent of novel laboratory techniques, like iTRAQ, that utilize an omics approach and allow a “global” approach for the detection of ideal biomarkers, may overcome the disadvantages of the conventional methods. Moreover, the combination of imaging and biofluid biomarkers regarding their prognostic role in TAI has not been studied in-depth, and future research should be performed to analyze whether such a combination could have a prognostic significance.

The majority of imaging biomarkers can be further analyzed in many hospitals and institutions since contemporary software can frequently support these novel techniques and is already utilized in neurooncology, stroke neuroimaging, and other fields of neurological research. Hence, it may be possible to conduct neuroimaging studies on patients with TAI in future studies and explore the spectrum of their diagnostic and prognostic potentials.

Still, despite significant progress in detection and diagnosis, the prognosis of patients with severe TAI remains poor. Moreover, the current therapeutic alternatives are limited and are focused on the prevention of secondary injuries such as brain hypoxia, intracranial hypertension, and development of hematoma, similar to the management principles of focal TBI. The advent of newer technologies providing further insights into the pathophysiology of secondary injury may lead to the development of novel interventions that will prevent or limit the acute and long-term consequences of axonal damage.

## Data Availability

Not applicable.
